# Ketorolac Loaded Poly(lactic-co-glycolic acid) Coating of AZ31 in the Treatment of Bone Fracture Pain

**DOI:** 10.3390/polym15102246

**Published:** 2023-05-09

**Authors:** Matteo Puccetti, Eleonora Cusati, Cinzia Antognelli, Maurizio Ricci, Valeria Ambrogi, Aurélie Schoubben

**Affiliations:** 1 Department of Pharmaceutical Sciences, University of Perugia, Via del Liceo 1, 06123 Perugia, Italy; 2 Department of Medicine and Surgery, University of Perugia, P.le L. Severi 1, 06129 Perugia, Italy

**Keywords:** magnesium alloy, solvent casting, AZ31 activation characterization, PLGA thermal behavior and mass loss, ketorolac release profile, primary human fibroblasts, primary human osteoblasts

## Abstract

Biodegradable metal alloys may be successfully used to support bone repair, avoiding second surgery commonly needed when inert metal alloys are used. Combining a biodegradable metal alloy with a suitable pain relief agent could improve patient quality of life. AZ31 alloy was coated using a poly(lactic-co-glycolic) acid (PLGA) polymer loaded with ketorolac tromethamine using the solvent casting method. The ketorolac release profile from the polymeric film and the coated AZ31 samples, the PLGA mass loss of polymeric film, and the cytotoxicity of the optimized coated alloy were assessed. The coated sample showed a ketorolac release that was prolonged for two weeks, which was slower than that of just the polymeric film, in simulated body fluid. PLGA mass loss was complete after a 45-day immersion in simulated body fluid. The PLGA coating was able to lower AZ31 and ketorolac tromethamine cytotoxicity observed in human osteoblasts. PLGA coating also prevents AZ31 cytotoxicity, which was identified in human fibroblasts. Therefore, PLGA was able to control ketorolac release and protect AZ31 from premature corrosion. These characteristics allow us to hypothesize that the use of ketorolac tromethamine-loaded PLGA coating on AZ31 in the management of bone fractures can favor osteosynthesis and relief pain.

## 1. Introduction

Some inert metal alloys, such as 316L steel, titanium and cobalt-chromium alloys, are used as permanent implants, allowing for the repair of damaged bones [1]. However, in recent years, attempts have been made to replace these alloys with biodegradable ones. In particular, magnesium alloys have several interesting properties, such as similar mechanical properties to those of human bones, cytocompatibility and in vivo degradation [2,3]. Among the various magnesium alloys on the market, this study focused on the AZ31 alloy characterized by the following composition (wt.%): 2.89 Al, 0.92 Zn, 0.05 Mn, 0.01 Si, 0.002 Cu, 0.001 Ni, 0.004 Fe and Mg to reach 100%. Compared to other magnesium alloys, AZ31 has a low Al content, good mechanical properties (increased strength and plasticity), fair corrosion resistance conferred by the Al, and is biodegradable [4,5]. Even though AZ31 alloy shows a higher resistance to corrosion with respect to Mg, its degradation in a physiological medium is still too fast. The degradation products of Mg alloy have been shown to be safe [6], but a too-rapid release of hydrogen gas and OH^−^, and the consequent effect of a rise in pH, makes the environment incompatible with surrounding tissue [7]. Gas production is an important factor that can also alter bone remodeling processes [8]. Furthermore, this rapid degradation could cause implant instability and failure if healing is not advanced to a sufficient degree to progress on its own [9,10].

Surface modifications of magnesium alloys can increase their resistance to corrosion, and in particular, the coating of the alloy can considerably improve the degradation times and therefore its biocompatibility [4]. It has been observed that the coating of the alloy with organosilanes [11], Mg-Al Layered Double Hydroxide (LDH) [12], plasma electrolytic oxidized/polycaprolactone (PEO/PCL) [13], plasma electrolytic oxidized/poly(l-lactide) (PEO/PLLA) [14], and poly(lactic-co-glycolic) acid (PLGA) [15,16] prolongs degradation times and favors the growth of bone tissue. PLGA is of particular interest for the coating of the alloy since it is an FDA-approved polymer widely used for medical and pharmaceutical applications. For instance, it has been employed to produce several drug delivery systems, such as microparticles, nanoparticles and in situ forming depot systems [17,18,19,20]. The interest of using PLGA to coat AZ31 alloy is twofold since, in addition to slowing down the degradation of the alloy, it can be used to control the release kinetics of active pharmaceutical ingredients (APIs). In the context of bone fractures, pain is a relevant problem, especially for rib fractures for which pain relief is the most important therapeutic act. For instance, non-steroidal anti-inflammatory drugs (NSAIDs) such as ketorolac and pain relief APIs such as paracetamol are administered parenterally [21,22]. The availability of an implant able to locally release the API could have significant advantages, such as (i) enabling effective local drug concentration with more effective relief from musculoskeletal pain, (ii) reducing drug side effects in the other body districts, and (iii) improving patient compliance. Ketorolac tromethamine is one of the NSAIDs used to achieve pain relief from bone fractures and surgery. As with other NSAIDs, it is responsible for several side effects and may inhibit osteoblast growth, correct mineralization, and bone organization. In particular, Ho et al. showed that 6 weeks of ketorolac treatment (4 mg/kg) in rabbits slowed the differentiation process during endochondral ossification, 2 weeks of treatment reduced bone union to some extent, and 2–4 weeks of treatment further reduced the mineralization of newly formed bone. Consequently, ketorolac can affect osteoblast proliferation and/or differentiation [23]. The optimization of the ketorolac tromethamine release profile could prevent cytotoxic effects while granting effective pain relief.

The main aim of this work was to produce a PLGA coating for the AZ31 alloy in order to increase its resistance to corrosion and decrease its cytotoxicity (which may be the result of a rapid production of degradation products). In addition, the presence of ketorolac tromethamine in the PLGA coating can improve patient compliance, avoiding the repeated administration of NSAIDs and allowing for its local release to relieve bone fracture and post-operative pain.

PLGA was successfully used to obtain an AZ31 coating loaded with ketorolac tromethamine. The API release, PLGA biodegradation and cytotoxicity studies evidenced the advantageous features of this coated AZ31 alloy over the uncoated one. This sample can be further studied to assess its potential for promoting osteosynthesis during bone fracture repair, with the advantage of avoiding a second surgery for its removal thanks to its biodegradation. Moreover, the ketorolac tromethamine loaded in the PLGA coating will provide local pain control.

## 2. Materials and Methods

### 2.1. Materials

Poly(lactic-co-glycolic) acid (PLGA, Resomer RG 503 H, lactic acid:glycolic acid 50:50, Mw 24,000–38,000 Da), methylene chloride (DCM), sodium chloride, sodium bicarbonate, potassium chloride, dibasic potassium phosphate trihydrate, magnesium chloride hexahydrate, hydrochloric acid, calcium chloride, sodium sulfate and tris(hydroxymethyl)aminomethane were purchased from Sigma-Aldrich (Milan, Italy). Ketorolac tromethamine was a gift from Recordati S.p.A. (Milan, Italy). Magnesium (Mg) alloy AZ31 (Zn 1%, Al 3%, Mg 96%) was purchased from GoodFellow (Hamburg, Germany). Sodium hydroxide was bought from Fluka Biochemika (Milan, Italy). Silicon rubber Silplay 184 was purchased from Prochima S.r.l. (Colli al Metauro, Italy). Neubauer counting chamber and Minisart NML filters (porosity 0.2 μm) were obtained from Thermo Fisher Scientific (Waltham, MA, USA). Ultrapure water (ρ = 18.3 MΩ cm@25 °C) was obtained from Synergy^®^ UV Water Purification System (Millipore Sigma, Merck KGaA, Darmstadt, Germany).

All chemicals and solvents were of analytical grade and were used without further purification.

### 2.2. Ketorolac Tromethamine Stability

Ketorolac tromethamine stability at 37 °C in simulated body fluid (SBF) was evaluated. SBF was prepared consistent with the paper by Marques et al. [24]. In particular, 500 mL of ultrapure water was introduced in a 1L volumetric flask and heated by magnetic stirring (240 rpm) to 37 °C. Once the temperature was reached, the components of the solution reported in Table S1 were progressively added one at a time following the addition order indicated in Table S1.

The solution was maintained under magnetic stirring (240 rpm) and at 37 °C for the duration of the whole process, and the successive ingredient was added only when the previous one was completely solubilized. Hydrochloric acid was added progressively a few milliliters at a time in order to monitor the pH of the solution and reach a value of 7.4. Attention had to be paid to avoid salt precipitation that could have been provoked by an inappropriate pH level, making the solution unusable. Once all the components were added, the volumetric flask was brought to volume with ultrapure water.

Ketorolac tromethamine was solubilized in SBF (20 μg/mL) and incubated at 37 °C. At predetermined timepoints (0, 24 and 96 h), an aliquot of the solution was withdrawn and analyzed by UV spectrophotometry at 322 nm (UV–vis Agilent 8453 Spectrophotometer, Agilent, Germany). The regression curve obtained for ketorolac tromethamine quantification was in the concentration range of 2.5–25 μg/mL and was characterized by good linearity (r^2^ = 0.99493). Ketorolac tromethamine was stable in SBF at 37 °C for the period investigated since its concentration was not modified.

### 2.3. Coating of AZ31 Alloy

Mg alloy AZ31 samples were soaked in a sodium hydroxide solution (20% *w*:*v*) heated at 60 °C and left under magnetic stirring (240 rpm) for 90 min. Samples were successively rinsed with ultrapure water and left to dry at room temperature. Lastly samples settled on a glass Petri dish were placed in an oven for 30 min at 80 °C, then for another 30 min at 120 °C. Samples were left in the oven while the oven heated up from 80 to 120 °C. Non-treated and treated Mg alloy AZ31 samples (referred as non-activated and activated) were analyzed by Raman spectroscopy and scanning electron microscopy (SEM).

A WITec Alpha300 RA confocal Raman microscope (WITec, Ulm, Germany) equipped with a diode-pumped solid-state laser module at 532 nm was used. Spectra were acquired at 10 mW source power, 60 accumulations, and 2 s integration time. Two-dimensional mapping of the treated alloy sample was performed at 100× magnification, tracking the OH stretching band on a 71 × 46 µm area at a resolution of 150 points/line × 150 lines/image and at 6.6 s/line scan speed, 0.5 s/line retrace speed, and 0.01 s integration time.

Non-activated and activated AZ31 samples were observed using SEM (LEO 1525 equipped with a GEMINI column, Zeiss, Oberkochen, Germany) after placing the samples onto an aluminum specimen stub covered with a double-sided adhesive carbon disc. Before imaging, samples were sputter coated with chromium (100 mA, 24 s, 8 nm thickness) (Quorum Q150T ES East Grinstead, Great Britain).

A polymeric film with 5% (*w*:*w*) ketorolac tromethamine was prepared using the solvent casting technique. To ensure that the solvent did not leak from the edges of the sample, AZ31 samples were introduced in a silicone rubber mold (Silplay 184) provided with 1 cm^2^ holes. PLGA was weighed (0.5 g) and solubilized under a fume hood into 5 mL of DCM. Once the polymer was solubilized, ketorolac tromethamine was added to the solution by stirring at 200 rpm for about one minute. Then, 300 μL of the suspension was dropped onto a 1 cm^2^ AZ31 sample that was previously placed in the mold, and this was left under a fume hood for 24 h to ensure complete evaporation of the organic solvent.

For the in vitro cytotoxicity study, smaller AZ31 disks were employed. In particular, each disk had a surface of 0.28 cm^2^ and was coated with the equivalent amount of suspension containing 10% (*w*:*v*) PLGA and 5% (*w*:*w*) ketorolac tromethamine following the same above-reported procedure.

### 2.4. Polymeric Film Production

Blank polymeric films and ketorolac-tromethamine-loaded polymeric films were prepared using the solvent casting method. A DCM solution of PLGA (10% *w*:*v*) was used to obtain blank polymeric films. The solution was poured into a glass Petri dish (diameter 9.4 cm) and evaporated for 24 h under a fume hood. The film was successively cut using a scalpel into samples weighing 40 mg each. A DCM solution of PLGA (10% *w*:*v*) containing 5% (*w*:*w*) ketorolac tromethamine was employed to produce drug-loaded films. Analogously to the coating procedure of AZ31 sample, the suspension (300 μL) was poured into the silicone rubber mold and left under the fume hood for 24 h to evaporate the solvent. Finally, samples were recovered by removing the loaded films from the mold. Both blank and ketorolac-tromethamine-loaded polymeric films were stored in a desiccator until further use.

### 2.5. Polymeric Films and Polymeric Coated AZ31 Characterization

Dried loaded films were analyzed to determine drug practical content. Briefly, tromethamine-loaded polymeric film was incubated in NaOH 1N (5 mL) for 24 h under stirring, and the sample, properly diluted in SBF, was analyzed by UV spectrophotometry at 322 nm. The experiment was performed in triplicate, and the results are expressed as mean drug content ± standard deviation.

Film thickness and alloy–polymer adhesion homogeneity of polymer-coated AZ31 samples was determined by SEM analysis of film cross sections. Briefly, activated and non-activated PLGA-coated samples were sliced thin by using a scalpel and were positioned on carbon-tape-coated aluminum stubs. The samples were sputter-coated as described for non-coated alloys. EDX analysis was performed with a Bruker Quantax EDS 200 probe. PLGA film thickness was evaluated by averaging two SEM pictures by image analysis using ImageJ software. An average of 24 distance points between the alloy–polymer interface and film surface were acquired to build up the film thickness profiles as a function of the physical distance from the film center. The overall mean film thickness was also calculated.

To characterize the thermal behavior of the films, differential scanning calorimetry (DSC) was performed by using a DSC821e (Mettler Toledo, Greifensee, Switzerland) equipped with a refrigerated cooling system. The system was calibrated by an indium standard. In particular, the following samples were analyzed: ketorolac tromethamine, PLGA, PLGA film loaded with 5% ketorolac tromethamine and ketorolac tromethamine:PLGA physical mixture (5:100, *w*:*w*). Approximately 3 to 5 mg of each sample was weighed in aluminum pans and sealed. The samples were subjected to an initial heating cycle from 0 to 80 °C at 5 °C/min, in order to erase the polymer thermo-mechanical history, and then to a second heating cycle from 0 to 230 °C at 5 °C/min. The Tg and ketorolac-tromethamine-melting endotherms were determined from the second heating ramp [25]. Data were analyzed with STARe software (Mettler Toledo, Greifensee, Switzerland), and the results were expressed as the mean of the 2 independent measures.

### 2.6. In Vitro Release Study

The ketorolac tromethamine in vitro release study with loaded polymeric films and the polymeric-coated AZ31 samples was carried out at 37 °C in SBF. Each sample was introduced in 10 mL of SBF and incubated at 37 °C in static conditions. Aliquots (1 mL) were collected at predetermined time intervals (1, 2, 4, 6, 8, 24, 48, 72 h, 7 days and every two days thereafter) and analyzed by UV spectrophotometry at 322 nm to quantify ketorolac tromethamine. Aliquots were immediately replaced with fresh SBF heated to 37 °C. The in vitro release study was performed in triplicate, and the results were expressed as the mean ketorolac tromethamine percentage released ± standard deviation. SBF pH at the end of the in vitro release study was measured and compared to that of fresh SBF and that of SBF containing an uncoated AZ31 sample for the same duration as in the release study. Simultaneously, AZ31 disks were observed to evaluate their degradation.

### 2.7. Blank Polymeric Film Mass Loss

Blank polymeric films (40 mg) were incubated in 10 mL round-bottom vials containing SBF (3 mL) at 37 °C in static conditions [26,27]. Films were recovered after 5, 10, 20, 30 and 45 days of immersion, rinsed with ultrapure water, and dried for 48 h at 37 °C. Dried films were weighed, and mass loss was determined for three different films for each time point and expressed as mean ± standard deviation.

### 2.8. Cell Culture and Treatments

BSCL138 primary human fibroblasts (IZSLER, Brescia, Italy) were cultured in Eagle’s minimum essential medium (MEM, Thermo Fisher Scientific, Waltham, MA, USA) supplemented with 10% fetal bovine serum (FBS, Thermo Fisher Scientific, Waltham, MA, USA), penicillin (10,000 U/mL), streptomycin (10,000 μg/mL) and 25 μg/mL amphotericin B as an antifungal agent (Thermo Fisher Scientific, Waltham, MA, USA) [28]. Primary human osteoblasts were purchased from PromoCell (Heidelberg, Germany) and cultured in DMEM (Invitrogen, Paisley, UK) supplemented with 10% fetal calf serum (FCS, Invitrogen, Paisley, UK), antimycotic and antibiotics (Invitrogen, Paisley, UK) [29]. The cells were seeded at a density of 1.6 × 10^4^/cm^2^ in an appropriate volume of medium and incubated in a humidified atmosphere at 37 °C with 5% CO_2_. In experimental points, except for the control, cells were seeded over AZ31 or AZ31 + PLGA or AZ31 + PLGA + ketorolac or PLGA. Each AZ31 sample, sterilized by UV light, was round shaped and had a total surface area of 0.28 cm^2^, close to that of the 24-well plates where the cell culture assays were conducted. This size similarity considerably limits the free movement of the specimen inside the well. After 24 h (the time necessary to let cells adhere to the well surface), ketorolac was added to the appropriate wells at a concentration of 12 µg/mL and 150 µg/mL, and all wells were incubated for additional 72 h. Fibroblasts and osteoblasts were chosen because they represent examples of the types of cells that orthopedic implants would be in contact with during clinical use [30]. In particular, SCL 138 primary human gingival fibroblasts, apart from retaining characteristics common to most fibroblasts, represent bone lining cells.

### 2.9. Cell Viability Assay

Cell viability was evaluated by MTT assay [31]. Cell survival was calculated relative to untreated control cells, which were set to 100%.

## 3. Results and Discussion

### 3.1. Polymeric Coated AZ31 Characterization

**Figure 1:**
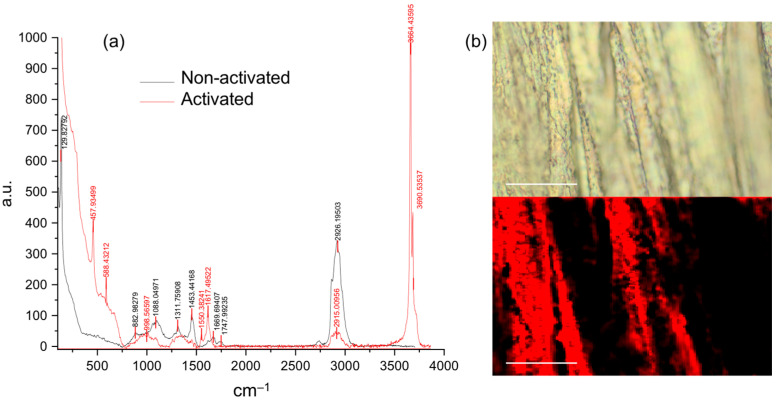
Raman spectroscopy characterization of AZ31. (**a**) Raman spectra of activated and non-activated AZ31 with relative main peaks and (**b**) 2D Raman mapping of activated AZ31 surface (above white-field image, below Raman mapping image). Analysis was performed at room temperature using a WITec Alpha300RA Raman microscope model (WITec, Ulm, Germany) equipped with a 532 nm laser source. Spectra were acquired at 10 mW source power, 60 accumulations, and 2 s integration time. The 2D mapping was performed at 100x magnification (bars 20 µm), tracking the OH stretching band at 3664 and 3690 cm^−1^ (in red) of the activated AZ31, on a 71 × 46 µm area, at a resolution of 150 points/line × 150 lines/image, and at 6.6 s/line scan speed, 0.5 s/line retrace speed, and 0.01 s integration time.

To facilitate the adhesion of the polymeric film, the AZ31 disks were activated to increase the number of hydroxyl groups present on the surface of the alloy. These groups were exploited to favor the binding that occurs between PLGA and AZ31. Activation was carried out in accordance with the procedure reported by Lin et al. [32]. The sodium hydroxide solution generates new hydroxyl groups on the surface of the alloy, while the heat treatment, especially the second step at 120 °C, stabilizes the layer produced [32]. Agarwal et al. [33] developed a PLGA multilayer coating to produce a corrosion-resistant and biocompatible surface on AZ31 for orthopedic applications. Similar to what has been done in this work, AZ31 was treated with sodium hydroxide to generate a layer of hydroxides. Since Resomer 503 H possesses free terminal carboxyl groups, it is likely that these are used in its bonding with the hydroxyl groups present on the AZ31 surface. To confirm AZ31 activation, Raman spectroscopy and an SEM/EDX analysis were performed. Figure 1 shows the Raman spectrum of the non-activated and activated AZ31 sample (a) and the 2D Raman mapping of the activated AZ31 surface (b).

Several differences were observed between the non-activated and the activated AZ31 spectrum (Figure 1a). In particular, the band at 1088 cm^−1^ ascribed to the MgO was strongly reduced in the activated AZ31 sample, while new bands corresponding to the hydroxyl stretching at 3664 cm^−1^ and 3690 cm^−1^ appeared in the activated sample [34,35]. These bands allow us to assert that hydroxyl groups were formed on the surface of the alloy. The 2D mapping performed that tracked the hydroxyl stretching showed a homogeneous distribution of OH groups (red color). The black regions correspond to areas of the sample that are out of focus, as evidenced in the white-field image (Figure 1b).

**Figure 2:**
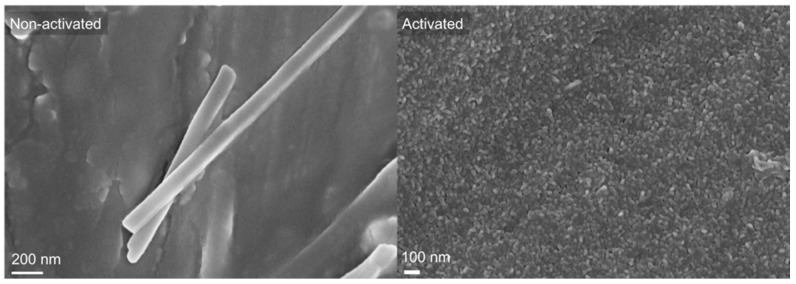
SEM photomicrographs of non-activated and activated AZ31 samples.

Besides Raman spectroscopy, SEM was performed, and photomicrographs evidenced the modification of the surface morphology (Figure 2).

The roughness of the activated sample was higher compared to that of the non-activated AZ31 sample where MgO crystals were observed. The surface roughness was similar to that observed by Lee et al. [36].

After activation, each sample was coated with the PLGA solution containing 5% (*w*:*w*) ketorolac using the solvent casting technique in the silicone rubber mold. The use of the mold allows the solution to deposit only on one of the two faces of the sample, preventing it from coating the other side of the sample. Once the DCM evaporated, the samples were removed from the mold, and the other side was covered using a cyanoacrylate surgical glue. Being coated on both faces, the corrosion of the sample will depend solely on the polymer coating degradation. To solubilize PLGA, DCM was chosen since it produces more robust samples and evaporates faster than chloroform. DCM can be completely removed, assuring the biocompatibility and therefore the absence of the cytotoxicity of the coating. Among other solvents able to solubilize PLGA, acetone evaporates even faster than DCM [37]. A too-fast evaporation would lead to the formation of an irregular and probably poorly adherent coating. It has also been shown that acetone and chloroform lead to the formation of films with more irregular porosities [38]. The solvent casting technique is one of the most used methods for the production of thin films since it is easy to achieve, and variables such as film thickness and size can be easily controlled. Several protocols have been reported in literature, and in general, the drying time is around 24–28 h [37].

**Figure 3:**
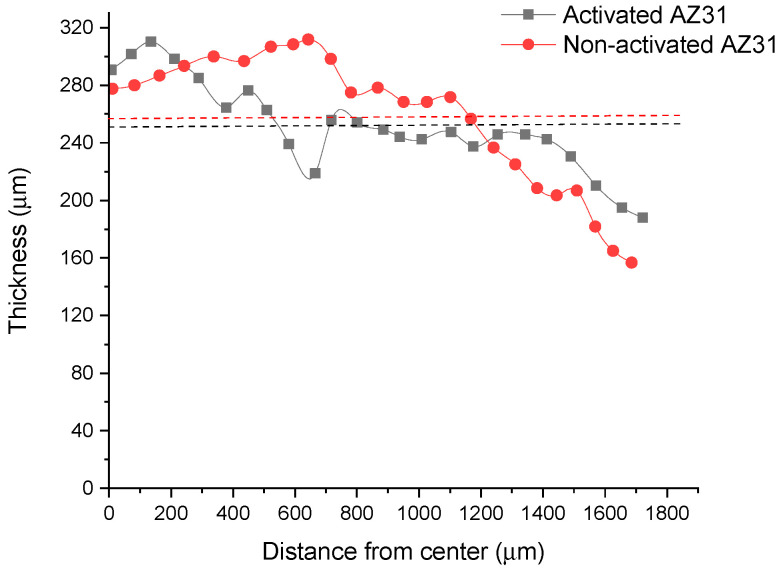
PLGA film thickness evaluation for activated and non-activated AZ31. Plots were generated by image analysis using ImageJ software. An average of 24 distance points were acquired from two different SEM images to build up the film thickness profiles as a function of the physical distance from the film center. Dotted lines represent the mean thickness values calculated for the PLGA films on activated (black) and non-activated (red) AZ31.

To evaluate the thickness of the film, SEM microscopy was used. Thickness is an important feature of the coating as it affects the ability of the film to encapsulate ketorolac tromethamine and protect the AZ31 alloy from corrosion. As observed in Figure 3, the PLGA film mean thickness was around 260 μm for both activated and non-activated AZ31 alloy, indicating a good reproducibility of the coating procedure. The film was thinner on the border of the alloy sample when compared to the center part, where the thickness was around 300 μm. Examples of the SEM images used to generate the plots of Figure 3 are reported in Figure S3.

**Figure 4:**
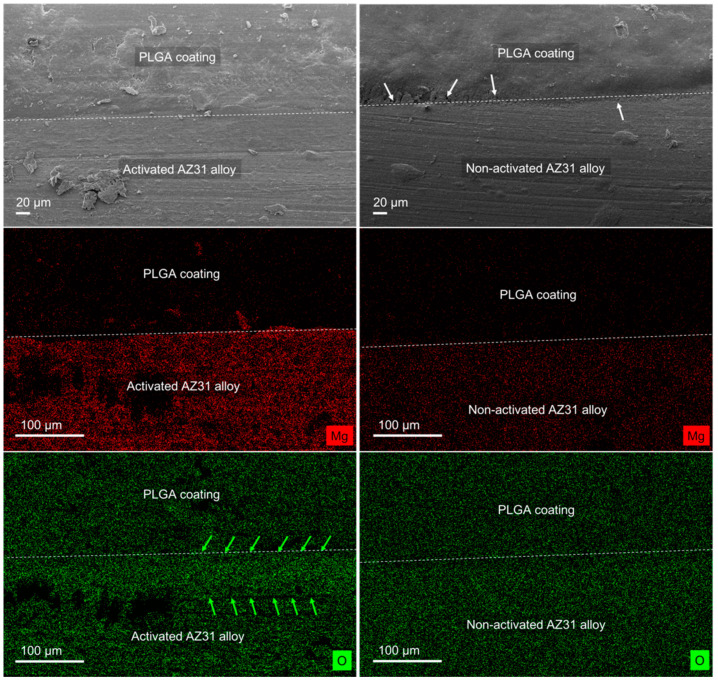
SEM photomicrographs of activated and non-activated AZ31 samples coated with PLGA. White arrows indicate pores observed at the alloy–polymer interface, which are signs of imperfect PLGA adhesion to non-activated alloy. EDX pictures of activated and non-activated AZ31 samples coated with PLGA evidencing the presence of Mg (red dots) corresponding to the alloy layer and oxygen (green dots) in both layers but at higher density at the surface of the activated AZ31 alloy (green layer between green arrows).

The SEM photomicrographs of the PLGA-coated alloy samples (Figure 4) allowed us to distinguish between AZ31 and the polymeric coating. In addition, from the analysis of the non-activated and activated alloy, a better adhesion of PLGA was identified for the activated AZ31. In fact, pores (indicated with white arrows in Figure 4) were observed on the non-activated AZ31 sample coated with PLGA, while a perfect continuity between the activated AZ31 and the PLGA was evidenced. EDX analysis was performed on the same samples to reveal the presence of the Mg and O elements. Mg localization was useful for discriminating the alloy layer from the polymeric coating, whereas oxygen was distributed on both layers. However, the oxygen density confirmed the higher amount of oxygen (layer between green arrows) on the surface of the activated AZ31 sample due to the presence of numerous OH groups (Figure 4). The OH groups, already identified using Raman spectroscopy, led to higher bonding forces with the copolymer, resulting in better polymer adhesion.

### 3.2. Differential Scanning Calorimetry

**Figure 5:**
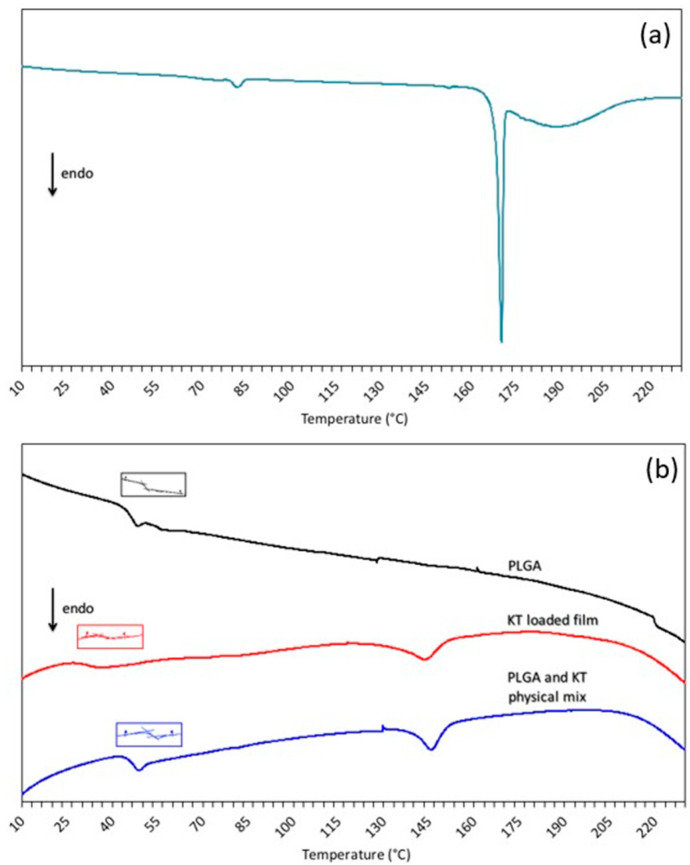
Thermograms of (**a**) ketorolac tromethamine and (**b**) PLGA, ketorolac tromethamine loaded PLGA film and ketorolac tromethamine: PLGA physical mixture. The reported thermograms correspond to the second heating run. Ketorolac tromethamine melting point and polymer Tg were determined on the second heating run.

Figure 5 shows the DSC data of (a) the ketorolac tromethamine and of (b) the PLGA, the ketorolac tromethamine loaded PLGA film and, the ketorolac tromethamine:PLGA physical mixture.

Ketorolac tromethamine shows an initial endothermic event at around 80 °C that can be ascribed to a loss of adsorbed water, while the endothermic peak at 170 °C corresponds to the melting of the ketorolac salt [39,40]. A third endothermic event can be observed in the temperature range of 180–210 °C associated with the decomposition of ketorolac tromethamine [39].

PLGA Tg, determined on the second heating ramp, can be observed in Figure 5b, and their values were reported in Table 1.

The raw PLGA Tg value matches the temperature already reported in the literature for the same polymer [41]. As evidenced in Table 1, the PLGA Tg of the physical mixture was not modified. On the contrary, the PLGA glass transition temperature of the ketorolac loaded film was significantly depressed. The ketorolac tromethamine exerted a noticeable plasticizing effect, with a decrease of about 14 °C from the Tg. Consequently, PLGA is in a rubbery state at body temperature. The plasticizing effect can be explained by the molecular dispersion of ketorolac in the polymeric network. However, at 5% (*w*:*w*) loading, the opacity of the film indicates that ketorolac tromethamine is not completely miscible with the polymer [25]. The presence of an endothermic event at ~145 °C for both the loaded film and the physical mixture suggests that some ketorolac crystals melt at a lower temperature with respect to raw ketorolac. This lowering can be ascribed both to the small dimensions of the ketorolac crystals and to the significant physical interactions between the polymer and ketorolac tromethamine. An active pharmaceutical ingredient melting point depression has already been reported in the literature with polymers such as polyethylene glycol and poly(vinylpyrrolidone) [42,43]. It has also been observed that melting lowering is more evident when the polymer is molten or in a rubbery state [42,43]. In fact, these polymer states favor interactions between the polymer and the active pharmaceutical ingredient, lowering its melting point. As already stated, no difference in melting temperature depression was observed between the physical mixture and the loaded film, indicating that the solvent casting method used to process the film was not responsible for the lowered temperature. Thus, the effect of water hydration in vitro and in vivo (not investigated in this study) should not be forgotten, since it has been demonstrated that it lowers the PLGA glass transition temperature [41].

### 3.3. In Vitro Release Study and Polymer Mass Loss

Having established the correct adhesion of the polymeric film, the amount of ketorolac tromethamine to be loaded into the polymer solution was selected. The amount of ketorolac must be safe for the cells surrounding the AZ31 implant and must be released with the appropriate kinetics, allowing for bone fracture and post-operative pain relief. Sinha et al. prepared biodegradable microspheres with ketorolac for parenteral administration using a ketorolac:PLGA ratio of 1:20 [44]. In this work, the same ratio was selected, while a ratio of 1:10 of polymer and DCM was used, as suggested by Manson et al. [37].

**Figure 6:**
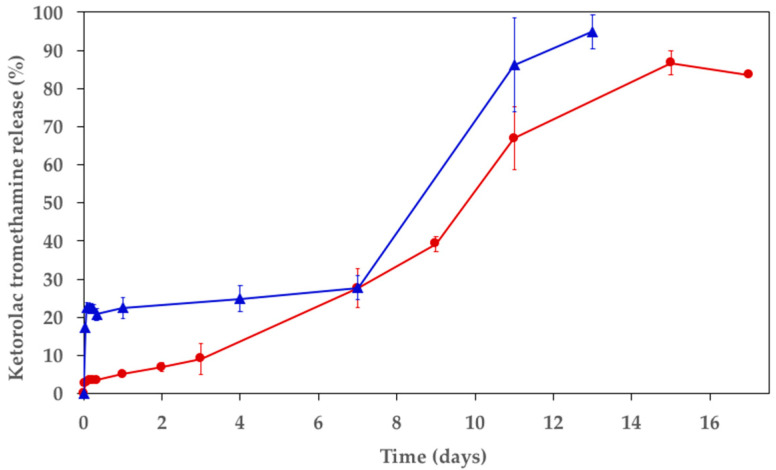
Ketorolac tromethamine release profiles from PLGA film (blue) and PLGA coated AZ31 alloy (red).

Ideally, the release of ketorolac tromethamine should be sustained for about two weeks, since a significant pain level lasts about fifteen days after a bone fracture [45]. However, prolonged administration at high concentrations has been shown to cause side effects or interfere with the healing of the bone [46,47,48]. The recommended dose of ketorolac tromethamine is 10 mg orally every 4–6 h, up to a maximum of 40 mg per day. Using AZ31, local administration will be guaranteed so that the amount of drug needed can be significantly lower, and the systemic exposure will be strongly limited. Ketorolac tromethamine release can therefore be extended beyond 5 days, which is commonly considered as the optimal duration for limiting adverse events following oral or parenteral administrations [49]. Polymeric film practical content coincides with the theoretical tromethamine loading of 5% (*w*:*w*). The ketorolac tromethamine in vitro release profiles from the polymeric film and the coated AZ31 alloy are shown in Figure 6. It is possible to notice that an initial burst release characterized the polymeric film (Figure 6, blue curve), with 17 and 22% released after the first hour and at 8 h, respectively. After a 1-week incubation in SBF, 40% of the ketorolac tromethamine was released, finally reaching 91% on the thirteenth day. The results obtained with the Resomer RG 503 H confirm that the release kinetics are compatible with the one desired. Since both faces of the polymeric film were exposed to the SBF, the release kinetics from the coated AZ31 were expected to be prolonged as only one side was exposed to the fluid. In fact, the ketorolac release was slower when the film was used to coat the AZ31 sample (Figure 6, red curve), while the profile was analogous to that of the sole PLGA film. In particular, no significant burst release was observed, and release lasted longer, reaching 80% after 15 days. During the in vitro release study, it was necessary to centrifuge the samples at the 72 h timepoint since some AZ31 debris were observed (Figure S1). Since the release was not complete, the causes of this behavior were investigated. When incubating a sample of AZ31 alloy for 15 days in SBF containing ketorolac tromethamine at 37 °C, a decrease in ketorolac concentration was observed. On the contrary, the ketorolac concentration was not modified when incubated in the same conditions but without AZ31. An additional assay was performed incubating ketorolac tromethamine in SBF that contains the degradation products of AZ31 but that was centrifuged to eliminate the AZ31 fragments produced during its corrosion. In this case, no decrease in the drug concentration was identified either. It was hypothesized that ketorolac tromethamine may form a complex with the AZ31 debris that originated from the alloy degradation, hindering drug quantification.

Along with the release kinetics, the SBF pH was measured to evaluate the effect of AZ31 degradation on its pH. As expected, the pH of SBF alone or containing drug loaded PLGA film was maintained for the entire duration of the study. On the contrary, the AZ31 alloy progressively increased SBF pH (Figure S2), owing to the alloy’s tendency to release OH^-^ and Mg^2+^ ions during its corrosion [50].

**Figure 7:**
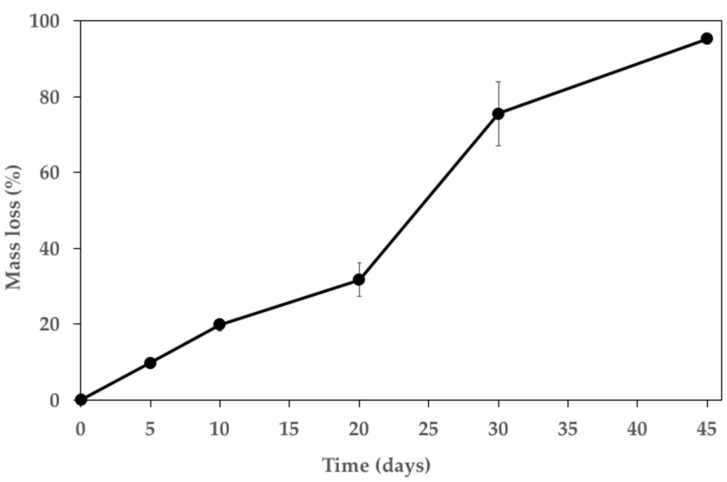
Mass loss profile pf PLGA film incubated in SBF at 37 °C.

Figure 7 shows the polymer film mass loss profile in SBF at 37 °C. For the first 20 days of immersion, the mass loss increased linearly by 10%, while for the 10 successive days (from day 20 to day 30), the degradation kinetics increased. Finally, the last part of the curve shows a slowdown of the mass loss. The mass loss was 75% after 30 days and reached up to 95% after 45 days of immersion. After 5 and 10 days, the film was almost intact and rigid, while it was slightly elastic after 20 days and became sticky and of poor consistency after 45 days. The PLGA mass loss profile was consistent with data already reported in the literature for the same polymer [26]. The differences observed can be ascribed to the diverse morphology of the polymer sample (i.e., film vs. microparticles). Besides its role in controlling ketorolac tromethamine release, the polymer degradation profile is compatible with a protective effect of AZ31 against corrosion. In fact, PLGA will limit AZ31 sample exposure to biological fluids, lowering its biodegradation and maintaining its mechanical properties for the duration needed for fracture repair.

### 3.4. AZ31 Cytotoxicity in Human Fibroblasts and Osteoblasts

It is of paramount importance to evaluate the in vitro cytotoxicity of AZ31 and ketorolac tromethamine prior to proposing their use in vivo. In the literature, this magnesium alloy is known to be a good candidate as biodegradable material for osteosynthesis [8,50,51]. Ketorolac tromethamine, on the other hand, was approved by the FDA in 1990 and is widely used to alleviate post-operative pain, particularly in the case of bone fractures [52]. However, at relatively low concentrations, ketorolac has demonstrated cytotoxicity that could hinder osteosynthesis. In fact, ketorolac tromethamine inhibits the production of prostaglandins, which are also responsible for the promotion of cell growth [53,54]. Therefore, given that the role of the AZ31 alloy is to promote osteosynthesis while ketorolac tromethamine treats fracture pain, it is imperative not to hinder this fundamental process for bone healing.

**Figure 8:**
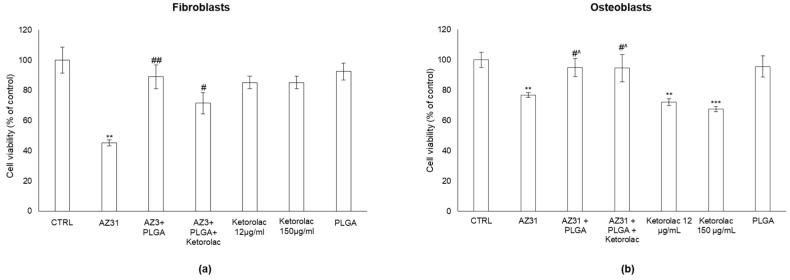
AZ31-induced cytotoxicity is prevented by PLGA in human fibroblasts and osteoblasts. Cell viability was measured by MTT assay in human (**a**) fibroblasts and (**b**) osteoblasts non-exposed (CTRL) or exposed to the different indicated agents. Exposure was performed for 72 h. Data report the means of three separate experiments performed in triplicate, and error bars represent the standard deviation (SD) of the mean. ** *p* < 0.01 and *** *p* < 0.001 significantly different from control unexposed cells. # *p* < 0.05 and ## *p* < 0.01 significantly different from AZ31-exposed cells. ^ *p* < 0.05 significantly different from ketorolac-exposed cells.

Figure 8 shows the effect of uncoated AZ31, PLGA-coated or PLGA/ketorolac-coated AZ31, ketorolac tromethamine and PLGA on human fibroblasts (Figure 8a) and osteoblasts (Figure 8b) compared with untreated cells (control).

As shown, uncoated AZ31 significantly decreases the viability of both fibroblasts and osteoblasts, resulting in a marked cytotoxicity to cells. This is in line with the results from other cell models in other species [55]. More importantly, neither PLGA-coated nor PLGA/ketorolac-coated AZ31 significantly affected cell viability in either model, indicating that the presence of PLGA prevents AZ31-induced cytotoxicity. Interestingly, ketorolac in osteoblasts, the more reliable model for implants with AZ31, induced a significant decrease in cell viability at both doses and was not cytotoxic when combined with PLGA-coated AZ31, thus further demonstrating the effective protective role of PLGA. PLGA was safe for both cell lines, as was expected for this FDA-approved polymer. PLGA was therefore able to mitigate the cytotoxicity of AZ31 related to its corrosion and that of ketorolac tromethamine on osteoblast by controlling its release rate. Therefore, PLGA coating has a dual role: (i) controlling tromethamine release, and (ii) slowing down AZ31 biodegradation.

## 4. Conclusions

AZ31 alloy was successfully coated with ketorolac tromethamine-loaded PLGA film. AZ31 activation has been proven to be essential for the correct adhesion of the polymer to the alloy. PLGA Resomer RG 503 H was characterized by a biodegradation profile suitable for controlling ketorolac tromethamine release for 15 days, while its complete degradation was obtained after 45 days. AZ31 alloy will therefore be in part protected from the exposure to the biological fluids, slowing down its corrosion process. This will decrease the loss of AZ31 mechanical properties, allowing for bone fracture support and healing. Interestingly, the PLGA coating was also successful in improving the cytocompatibility of the alloy, suggesting that it is safe for both human osteoblast and fibroblast cell lines. In addition, the ketorolac tromethamine cytotoxicity observed in osteoblasts was mitigated once it was included in the polymer coating.

## Figures and Tables

**Table 1:** Glass transition temperature of PLGA, ketorolac tromethamine loaded PLGA film and ketorolac tromethamine:PLGA physical mixture.

Sample	Tg (°C)
PLGA RG 503 H	45.73
PLGA film loaded with 5% ketorolac tromethamine	31.93
Ketorolac tromethamine:PLGA physical mixture (5:100, *w*:*w*)	45.82

## Data Availability

Not applicable.

## Supplementary Materials

The following supporting information can be downloaded at: https://www.mdpi.com/article/10.3390/polym15102246/s1, Figure S1: AZ31 coated with PLGA after 72 h (first row) and one month (second row) of immersion. Despite the significant hydrolysis of the polymer film, it remained adherent to the alloy, slowing down its corrosion. Some AZ31 alloy debris (red arrows) can already be observed after 72 h; Figure S2: pH values of fresh SBF and SBF incubated at 37 °C in presence of ketorolac loaded PLGA film, coated AZ31 sample and uncoated AZ31 sample; Figure S3: SEM photomicrographs of activated and non-activated AZ31 coated with PLGA film; Table S1: Quali-quantitative composition of the simulated body fluid.
