# Supplementary material for: Ketorolac Loaded Poly(lactic-co-glycolic acid) Coating of AZ31 in the Treatment of Bone Fracture Pain
Source: Polymers (Basel). 2023 May 9;15(10):2246. doi: 10.3390/polym15102246 (PMC10223411; doi:10.3390/polym15102246)
Supplement: Supplementary file 1 [file polymers-15-02246-s001.zip › polymers-2318019-supplementary.pdf]

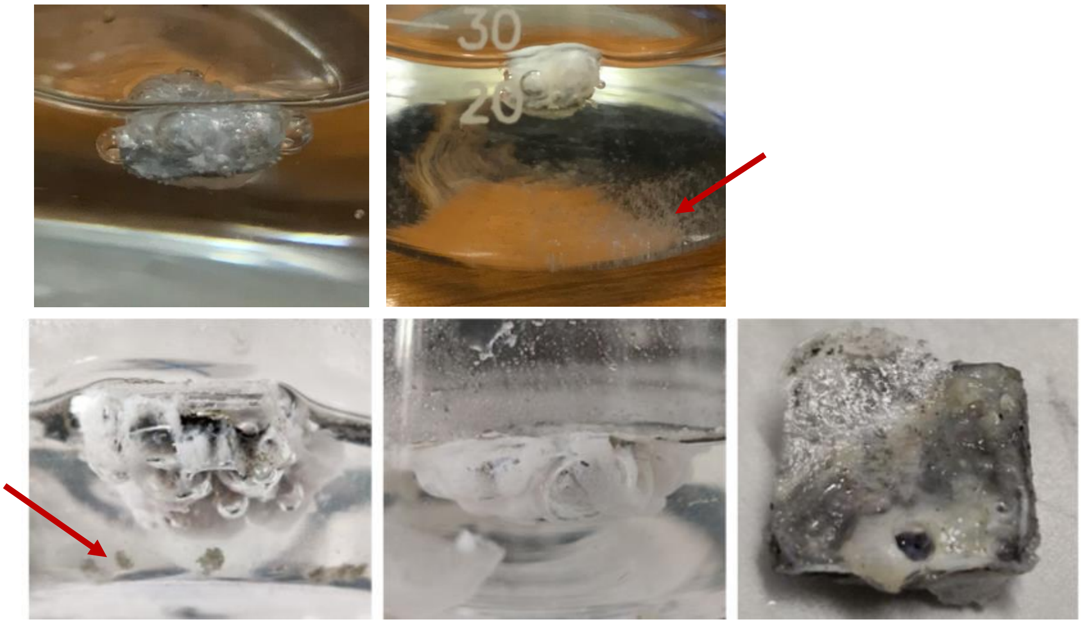

Figure S1: AZ31 coated with PLGA after 72 hours (first row) and one month (second row) of immersion. Despite the significant hydrolysis of the polymer film, it remained adherent to the alloy, slowing down its corrosion. Some AZ31 alloy debris (red arrows) can be observed already after 72 hours.

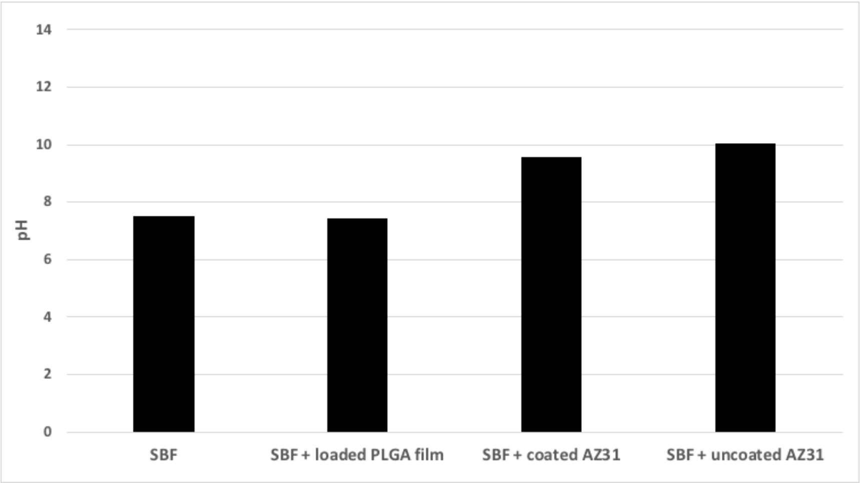

Figure S2: pH values of fresh SBF and SBF incubated at 37 °C in presence of ketorolac loaded PLGA film, coated AZ31 sample and uncoated AZ31 sample.

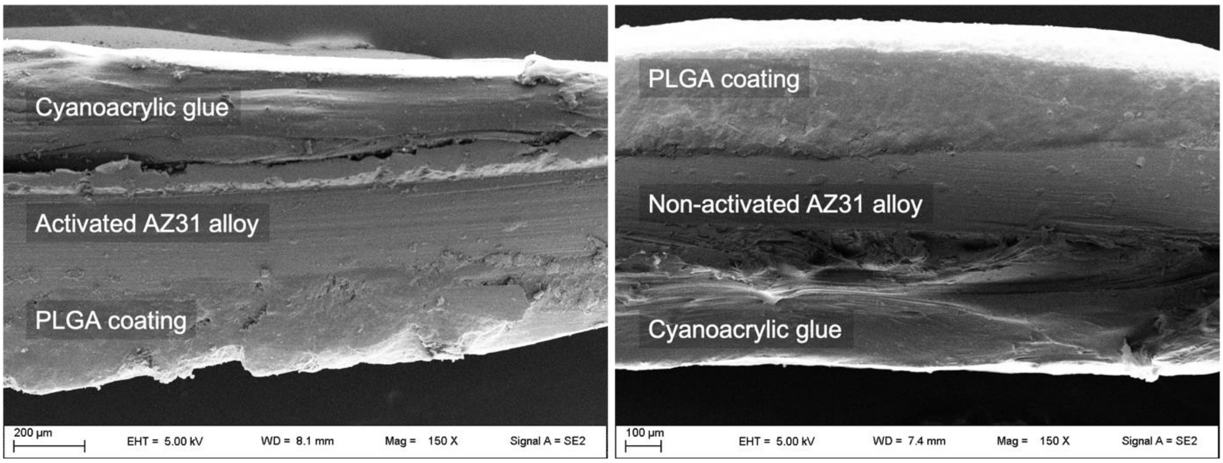

Figure S3: SEM photomicrographs of activated and non-activated AZ31 coated with PLGA film.

Table S1. Quali-quantitative composition of the simulated body fluid.

Addition order	Compound	Amount for 1L (g)
1	Sodium chloride	8.035
2	Sodium bicarbonate	0.355
3	Potassium chloride	0.225
4	Dibasic potassium phosphate trihydrate	0.231
5	Magnesium chloride hexahydrate	0.311
6	Hydrochloric acid 1M	39[1]
7	Calcium chloride	0.292
8	Sodium sulfate	0.072
9	Tri (hydroxymethyl) aminomethane	6.118

[1] Expressed in mL.
